# Prediction Model for Pancreatic Cancer—A Population-Based Study from NHIRD

**DOI:** 10.3390/cancers14040882

**Published:** 2022-02-10

**Authors:** Hsiu-An Lee, Kuan-Wen Chen, Chien-Yeh Hsu

**Affiliations:** 1 National Health Research Institutes—The National Institute of Cancer Research, 367, Shengli Road, North District, Tainan 704, Taiwan; hsiuanlee@nhri.edu.tw; 2 Department of Information Management, National Taipei University of Nursing and Health Sciences, Taipei 112303, Taiwan; 3 Master Program in Global Health and Development, College of Public Health, Taipei Medical University, Taipei 110, Taiwan

**Keywords:** prediction model, early screening, personal health, precision health, pancreatic prevention

## Abstract

**Simple Summary:**

Pancreatic cancer has been ranked seventh in the top ten cancer mortality rates for the past three year in Taiwan. It is one of the more difficult cancers to detect early due to the lack of early diagnostic tools. This is a population-based study from NHIRD. A higher performance pancreatic cancer prediction model has been established. This predictive model can improve the awareness of the risk of pancreatic cancer and give patients with pancreatic cancer a simpler tool for early screening in the golden period when the disease can still be eradicated.

**Abstract:**

(1) Background: Cancer has been the leading cause of death in Taiwan for 39 years, and among them, pancreatic cancer has been ranked seventh in the top ten cancer mortality rates for the past three years. While the incidence rate of pancreatic cancer is ranked at the bottom of the top 10 cancers, the survival rate is very low. Pancreatic cancer is one of the more difficult cancers to detect early due to the lack of early diagnostic tools. Early screening is important for the treatment of pancreatic cancer. Only a few studies have designed predictive models for pancreatic cancer. (2) Methods: The Taiwan Health Insurance Database was used in this study, covering over 99% of the population in Taiwan. The subset sample was not significantly different from the original NHIRD sample. A machine learning approach was used to develop a predictive model for pancreatic cancer disease. Four models, including logistic regression, deep neural networks, ensemble learning, and voting ensemble were used in this study. The ROC curve and a confusion matrix were used to evaluate the accuracy of the pancreatic cancer prediction models. (3) Results: The AUC of the LR model was higher than the other three models in the external testing set for all three of the factor combinations. Sensitivity was best measured by the stacking model for the first factor combinations, and specificity was best measured by the DNN model for the second factor combination. The result of the model that used only nine factors (third factor combinations) was equal to the other two factor combinations. The AUC of the previous models for the early assessment of pancreatic cancer ranged from approximately 0.57 to 0.71. The AUC of this study was higher than that of previous studies and ranged from 0.71 to 0.76, which provides higher accuracy. (4) Conclusions: This study compared the performances of LR, DNN, stacking, and voting models for pancreatic cancer prediction and constructed a pancreatic cancer prediction model with accuracy higher than that of previous studies. This predictive model will improve awareness of the risk of pancreatic cancer and give patients with pancreatic cancer a simpler tool for early screening in the golden period when the disease can still be eradicated.

## 1. Introduction

While targeted drugs are one of the treatments for cancer, there is still a lack of widely available and effective targeted drugs for pancreatic cancer. According to recent studies, pancreatic cancer does not have a specific molecular variant, as lung and breast cancers do, and researchers have even suggested that there may be more than one molecular variant in pancreatic cancer. Pancreatic cancer tends to have more non-specific symptoms than other diseases, which often leads to the initial diagnosis of other abdominal diseases, making initial treatment plans ineffective and delaying treatment. The lack of early diagnostic tools for pancreatic cancer results in poor overall outcomes [1].

Cancer has been the leading cause of death in Taiwan for 39 years, and among them, pancreatic cancer has been ranked seventh in the top ten cancer mortality rates for the past three years [2]. While the incidence rate of pancreatic cancer is ranked at the bottom of the top 10 cancers, the survival rate is very low. In Taiwan, the survival rate of pancreatic cancer patients is about 25.52% at one year, 9.22% at three years, 6.6% at five years, and 4.71% at ten years. Based on the number of new diagnoses and deaths each year, it is estimated that the incidence rate of the disease is generally close to the mortality rate [3,4].

Due to the absence of obvious disease features in the initial stage, and the fact that the tumor is located in the posterior abdominal cavity, pancreatic cancer is often diagnosed at an advanced stage [4]. According to the American Cancer Society’s Facts and Figures annual report, the average five-year survival rate for pancreatic cancer is 9%, with 37% of patients having an early localized disease and only 3% of patients having an advanced disease [5]. In 2019, a joint study by the Lancet and the Global Burden of Diseases (GBD) found that, between 1990 and 2017, the number of deaths, incident cases, and disability-adjusted life years (DALYs) of pancreatic cancer doubled worldwide. Therefore, there is an urgent need for a screening method and effective treatment strategy for the early detection of pancreatic cancer [6]. Surgery remains the ideal treatment for pancreatic cancer and is currently the only method considered to have a chance of curing pancreatic cancer, which significantly improves patient survival compared to other treatment options [7,8]. However, the success rate of surgery depends on the stage of the disease. If the tumor has invaded a major artery or has metastasized distantly, the chances of surgery for pancreatic cancer are low. The data show that only 10–15% of patients have a chance of undergoing radical surgery, with most patients having a tumor that is too large by the time it is discovered and the surrounding lymph nodes, blood vessels, and nerves have been eroded. In 2014, the International Study Group of Pancreatic Surgery (ISGPS) published a study stating that formal resection is highly discouraged if the cancer shows signs of erosion of the superior mesenteric artery [9].

The pre-cancerous nature of pancreatic cancer is not well understood in current studies, and evidence of its incidence, biomarkers, and natural history progression remain insufficient [10,11]; therefore, there is a great need for successful early detection markers when screening for pancreatic cancer in populations at higher risk. Epidemiological studies of clinical factors can be used to effectively reduce and screen high-risk groups [12,13].

A number of symptoms and diseases have been found to be associated with pancreatic cancer; for example, pancreatitis (idiopathic pancreatitis, alcoholic pancreatitis, hereditary pancreatitis, and febrile pancreatitis) is associated with a significantly increased risk of pancreatic cancer [14,15]. Compared to other inflammatory conditions, chronic pancreatitis has a relative risk (RR) or odds ratio (OR), which is one of the higher risk factors for pancreatic cancer [16,17]. 

T2DM is considered to be an early manifestation of asymptomatic pancreatic cancer and has been suggested as a potential early detection marker [18,19]. Annika Bergquist showed that patients with primary sclerosing cholangitis had a 37% risk of developing hepatobiliary malignancies within one year of diagnosis, a 161-fold increase in the risk of hepatobiliary malignancies, and a 14-fold increase in the risk of pancreatic cancer, as compared to those without prior cholangitis [20]. Thus, cholangitis is a significant risk factor for pancreatic cancer [21]. Clinical observations support the potential carcinogenic role of the hepatitis B virus (HBV) in pancreatic tumors [22,23]. According to the National Health and Nutrition Examination Survey (NHANES) epidemiological follow-up survey, people with periodontitis were found to be at higher risk of developing pancreatic cancer [24,25]. Another study showed a strong positive association between periodontal disease and pancreatic cancer [26]. Pancreatic cancer occurs predominantly in older patients, and only about 10% of patients that develop a tumor are under 50 years old, thus, the incidence rate increases rapidly with age [27].

Only a few studies have designed predictive models for pancreatic cancer. Limor Appelbaum et al. used a feedforward neural network and logistics regression, with an AUC of 0.71 for the training set and 0.68 for the validation set [28], in order to construct a model for the early assessment of pancreatic cancer. A case-control study by Aileen Baecker et al. developed a multivariate logistic regression model using 16 risk factors and patients’ symptoms in the 15 months prior to the diagnosis of pancreatic cancer. After matching the age and the gender of the two groups, the model showed that both the case and the control groups’ predictions achieved an AUC of 0.68 [29]. Alison P. Klein et al. developed a predictive model based on odds ratio (OR), including smoking, alcohol consumption, diabetes, obesity, family history of pancreatic cancer, and non-O ABO genotype. The subsequent results showed that the AUCs of the risk models were 58%, 57%, and 61% for non-genetic, genetic-only, and non-genetic and genetic factors, respectively [30].

## 2. Materials and Methods

### 2.1. Data Source

This study used data from the Taiwan Health Insurance Database (NHIRD) from 2000 to 2009. The NHIRD covers 99.98% of the population in Taiwan [31] and contains basic data files, outpatient and inpatient surgical, diagnostic, and medication information, treatment records, and other clinical detailed information. The dataset used in this study was published by NHI Taiwan and contains all of the data for 2,000,000 randomly sampled individuals from NHIRD. The subset sample was not significantly different from the original NHIRD sample in terms of gender and age distribution.

As the diagnostic data contained in NHIRD may only be a one-off diagnosis made for cancer examination, the experimental group excluded cases with less than two diagnoses of pancreatic cancer.

As metastasis was not a predictive target, only patients without any prior malignancy (cancer) were included in the experimental group. Ultimately, the time of the first diagnosis of pancreatic cancer in the experimental group was used as the study data, which incorporated the short-term historical medical information within one year before diagnosis date. The targeted control group had never had any malignancy in the entire database, and in order to avoid bias and inequity due to age and gender, the sample was matched according to the gender and age of the subjects in the experimental group. A one-to-three control ratio was used for the control group sample [32], thus, a total of 738 subjects were included in the experimental group and 2952 subjects in the control group.

### 2.2. Data Processing

The dataset was divided into a training set and a testing set in a ratio of 8:2. The training set was divided into an 80% training set for model training and a 20% validation set for model validation. The testing set was the external test data, which was used for the final performance testing of the model. As data imbalance may lead to model accuracy being compromised [33], the plain sampling approach was used during the training and validation phases, where a few types of data were randomly re-sampled to achieve a 1:1 balance. The Random Over Sampler suite, as provided by Python, used imbalanced learning to implement the plain sampling method by randomly re-sampling a small number of categories until a numerical balance was achieved, relative to the majority of categories. In order to ensure fairness of the external test data, the testing set was not involved in any significant factor checking or sampling process. 

Chi-squared testing was used to examine the relationship between the factors of the categorical variables and pancreatic cancer in this study to initially confirm the statistical significance of the factors. The Akaike information criterion (AIC) was used to identify the best factor combination for inclusion in the model training. 

### 2.3. Model Training

#### 2.3.1. Logistic Regression, LR 

Logistic regression [34] is a probabilistic non-linear regression model for multivariate analysis of the relationship between a binary outcome (Y) and multiple variables $\;\left(X_{1,}X_{2,}\ldots ,\;X_{3}\right)$. In contrast, linear regression aims to fit all data points into a straight line to predict a continuous value. In general, during linear regression, the equation is constructed directly on the target *Y* using the feature *X*. In logistic regression, a linear equation is constructed by taking the logarithm of the odds ratio and converting it to a sigmoid function with a probability range between 0 and 1, representing 0% and 100%, respectively, for the purpose of binary classification.

#### 2.3.2. Deep Neural Networks (DNN) 

DNNs [35] are neural-like networks with multiple hidden layers. While their architecture is similar to that of early perceptrons, the main differences are the deeper hidden layers, the greater variety of activation functions, and the generally better fitting effect. A single-layer perceptron can be regarded as a simple feedforward neural network, where $X_{i}$ is the input factor, $W_{i}$ is the weight value, and function is the activation function that simulates the structure of nerve cells in a living organism. However, as single-layer perceptrons are unable to learn more complex non-linear models, or provide multivariate outputs, they have been extended to deep neural networks.

#### 2.3.3. Ensemble Learning

Ensemble learning [36,37] refers to the systematic combination of multiple classifiers, and the aim of combining multiple classifiers is to produce a more powerful model. It is primarily used to improve the classification, prediction, and function approximation performance of a model or to reduce the likelihood of selecting a poorly performing algorithm.

**Figure 1:**
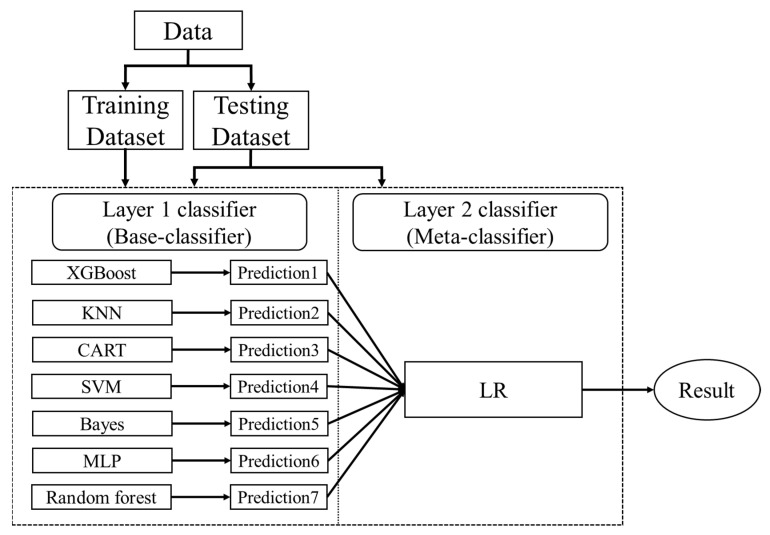
Stack ensemble learning model architecture.

Stacking is an ensemble learning approach that focuses on reducing bias by combining multiple prediction results. It consists of a two-tier structure, with the first tier being used to build multiple base classifiers, and the base classifiers of the first tier being combined by a meta-learner (logistic regression) in the second tier. Seven base classifiers were used in this study, including the following: Multi-Level Perceptron (MLP), Random Forest (RF), Support Vector Machine (SVM), K-Nearest Neighbor (KNN), Gradient Boosted Decision Tree (XGBoost), Classification and Regression Tree (CART), and Bayes. The Stack ensemble learning model architecture is shown in Figure 1. The stacking steps are as follows:

(1) Split the data into a training set and a testing set
(2) Split the training set by k-fold
(3) Train and predict until a prediction is available for each fold
(4) Combine a base model on the complete training set
(5) Use the model to make predictions on the testing set
(6) Repeat the above steps for the other base models
(7) Use all predictions from the base model as the learning features for the new model (meta-learners)
(8) Use the new model to make final predictions on the testing set

#### 2.3.4. Voting Ensemble

**Figure 2:**
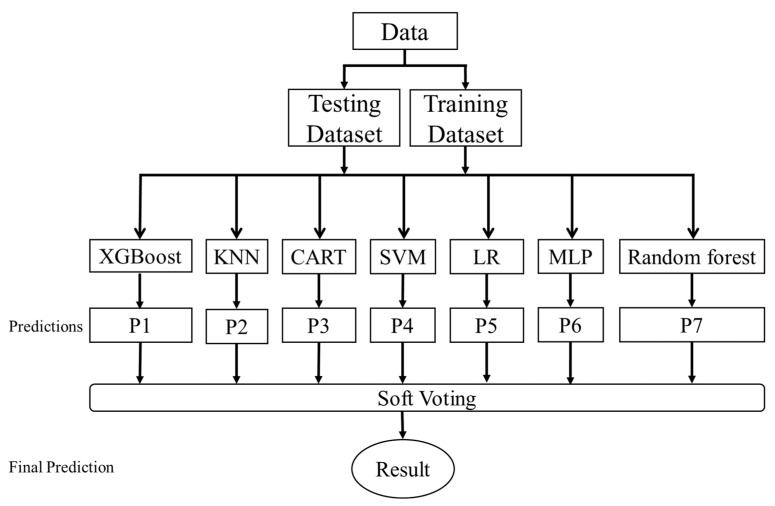
Voting ensemble model architecture.

The voting ensemble is an ensemble learning method that combines the predictions of several different models, also known as majority voting ensembles, which can be used for classification or regression tasks. The regression task calculates the average of the predictions of the models. The categorization task determines the final outcome by a majority vote of the predicted category results of each model. This study used seven models, including the following: MLP, LR, RF, SVM, KNN, XGBoost, and Classification Soft Voting, to sum and average the predicted class odds for each model. The voting ensemble model architecture is shown in Figure 2.

### 2.4. Model Development Environment

This study used SPSS 22 for data pre-processing, chi-squared independence checking, and sample matching; R language version 3.5.2 for AIC criterion checking, such as backward elimination in stepwise regression; Python version 3.7.3 for logistic regression (LR) [34], deep neural networks (DNN) [35], ensemble learning [36,37], voting ensemble model building, and visual mapping; Microsoft SQL Server 2014 was used for database retrieval and filtering. The ROC curve and a confusion matrix were used to evaluate the accuracy of the pancreatic cancer prediction models.

## 3. Results

A total of 3690 subjects were included in this study. The dataset was divided into a training set and a testing set in a ratio of 8:2. The training set (N = 2952) was divided into an 80% training subset (N = 2362) for model training and a 20% validation set (N = 590) for model validation. There were more male subjects present (53.9 vs. 46.1). Age was concentrated in the middle to old age group, mainly over 65 years of age (more than 55%). A total of 2952 subjects in the training set were identified as having risk factors associated with pancreatic cancer in the first stage. The risk factors identified in previous studies, including pancreatitis, diabetes, peptic ulcer, cholangitis, hepatitis, periodontal disease, sleep disorders, and fasciitis, were included in this study. Other factors were obtained from the short-term medical history of the pancreatic cancer patients in the study group over the period of one year. Finally, a total of 74 candidate factors were included in the follow-up factor validation. 

This study used three combinations of factors for model training. The first combination is the significant factors (*p*-value < 0.05), with a total of 32 factors (hereinafter referred to as the first factor combinations), including the following: abdominal pain (ICD-9 = 789), peptic ulcer, site unspecified (ICD-9 = 533), symptoms involving the digestive system (ICD-9 = 787), pancreatitis (ICD-9 = 577), diabetes mellitus (ICD-9 = 250), gastritis and duodenitis (ICD-9 = 535), disorders of function of the stomach (ICD-9 = 536), functional digestive disorders, not elsewhere classified (ICD-9 = 564), chronic liver disease and cirrhosis (ICD-9 = 571), general symptoms (ICD-9 = 780), gastric ulcer (ICD-9 = 531), cholangitis (ICD-9 = 576), calculus of kidney and ureter (ICD-9 = 592), duodenal ulcer (ICD-9 = 532), acute bronchitis and bronchiolitis (ICD-9 = 466), symptoms involving the head and neck (ICD-9 = 784), symptoms involving the respiratory system and other chest symptoms (ICD-9 = 786), acute nasopharyngitis (ICD-9 = 460), urticaria (ICD-9 = 708), cardiac dysrhythmias (ICD-9 = 427), other cellulitis and abscess (ICD-9 = 682), acute and subacute necrosis of the liver (ICD-9 = 570), hypertensive heart disease (ICD-9 = 402), heart failure (ICD-9 = 428), other disorders of pancreatic internal secretion (ICD-9 = 251), other forms of chronic ischemic heart disease (ICD-9 = 414), gout (ICD-9 = 274), neurotic disorders (ICD-9 = 300), essential hypertension (ICD-9 = 401), acute laryngitis and tracheitis (ICD-9 = 464), osteoarthrosis and allied disorders (ICD-9 = 715), and other and unspecified disorders of the back (ICD-9 = 724). The demography of the study group is shown in Table 1.

In order to streamline the number of factors to improve the convenience and reduce the complexity of the model, the next two combinations attempted to use more stringent statistical validity as a factor for the selection criterion. The purpose was to identify the key factors with truly high predictive power among the significant factors (first factor combinations).

The second factor combination used backward elimination in stepwise regression to select 19 factors with *p*-values of <0.05 from the first factor combinations, including the following: abdominal pain, peptic ulcer site unspecified, symptoms involving the digestive system, gastritis and duodenitis, disorders of function of the stomach, chronic liver disease and cirrhosis, general symptoms, cholangitis, pancreatitis, symptoms involving the head and neck, symptoms involving the respiratory system and other chest symptoms, urticaria, other cellulitis and abscess, acute bronchitis and bronchiolitis, cardiac dysrhythmias, acute and subacute necrosis of liver, diabetes mellitus, gout and functional digestive disorders not elsewhere classified. The third factor combination used backward elimination in stepwise regression to select nine factors with *p*-values of <0.001 from the first factor combinations, including the following: abdominal pain, peptic ulcer site unspecified, symptoms involving the digestive system, gastritis and duodenitis, disorders of function of the stomach, chronic liver disease and cirrhosis, general symptoms, cholangitis, and pancreatitis. The stepwise regression results is shown in Table 2:

This study constructed four models using three different sets of factors. The ROC curve has both validation and testing sets. While the validation set was used in the aforementioned chi-square and stepwise regression, it was not included in the model training. While the testing set was not used for factor selection or model training, it was used to simulate real data for testing.

### 3.1. Model Performance Comparison 

This study compared the performance of each model in the validation and testing sets by using three different sets of ROC curve factors, accuracy, sensitivity, and specificity.

#### 3.1.1. First Factor Combinations (32 Factors)

**Figure 3:**
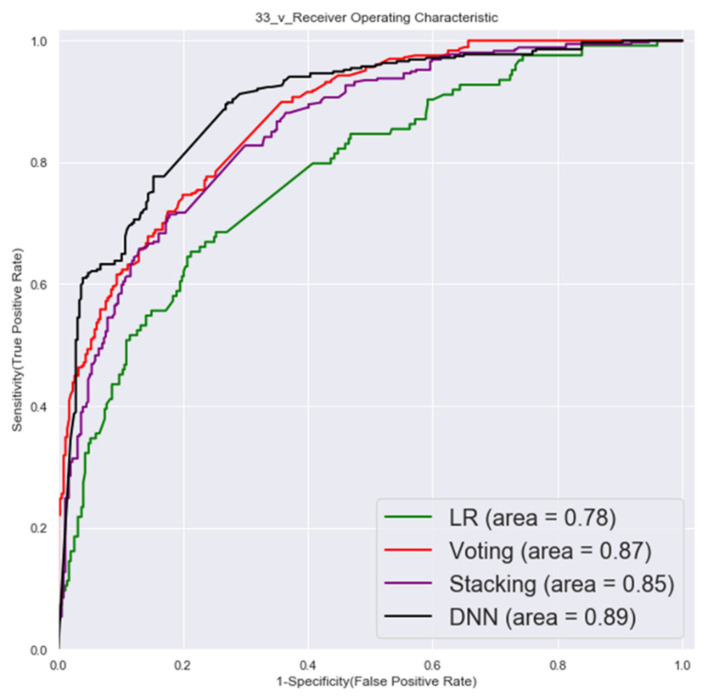
Validation set ROC (first factor combinations).

**Figure 4:**
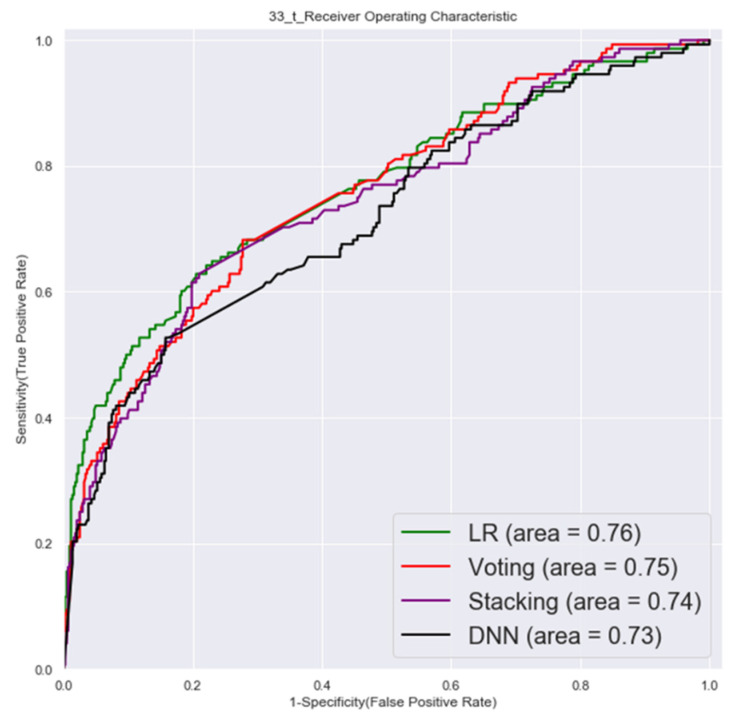
Testing set ROC (first factor combinations).

The ROC of validation set and testing by first factor combinations are shown in Figure 3 and Figure 4.

#### 3.1.2. Second Factor Combinations (19 Factors)

**Figure 5:**
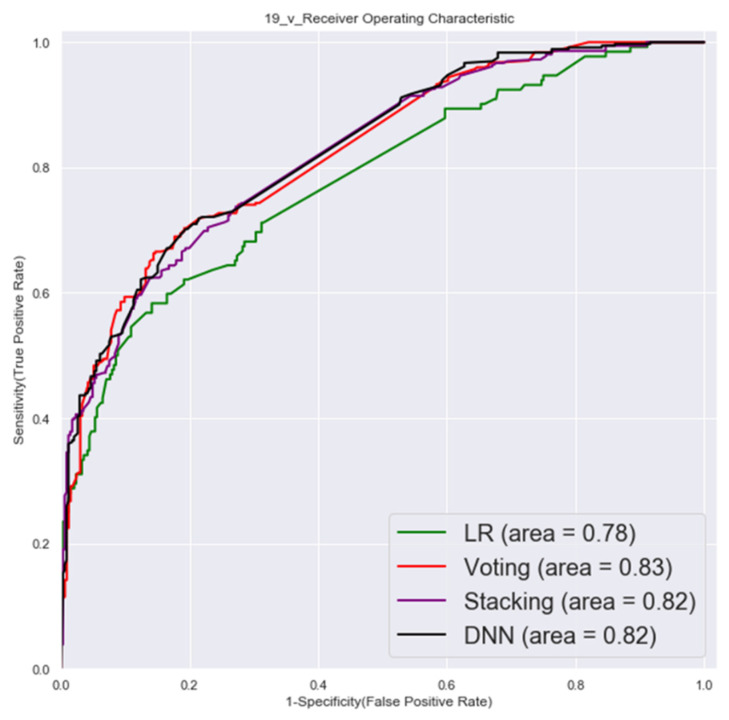
Validation set ROC (second factor combinations).

**Figure 6:**
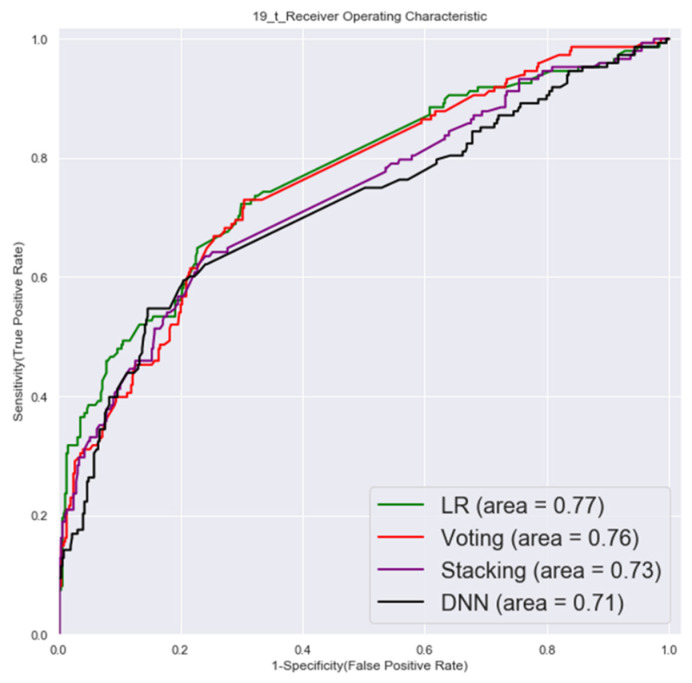
Testing set ROC (second factor combinations).

The ROC of validation set and testing by second factor combinations are shown in Figure 5 and Figure 6.

#### 3.1.3. Third Factor Combinations (9 Factors)

**Figure 7:**
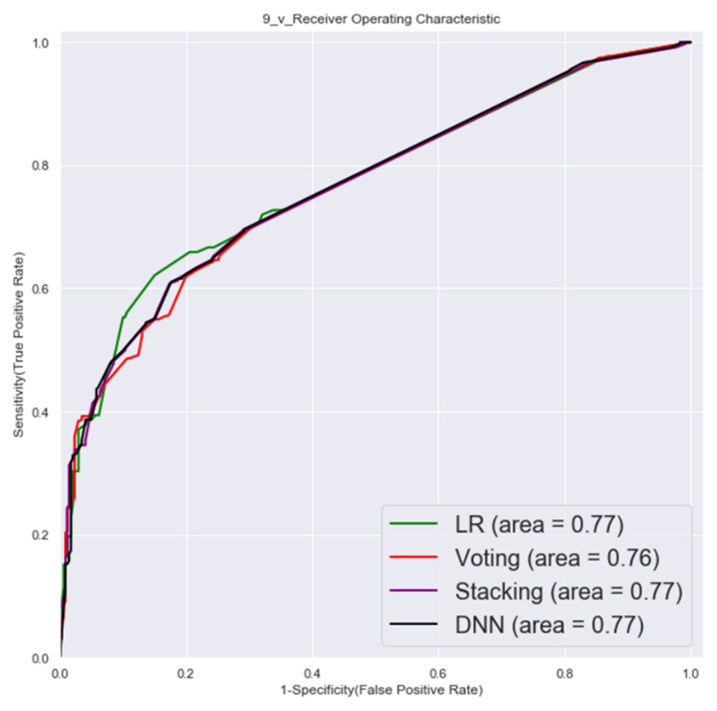
Validation set ROC (third factor combinations).

**Figure 8:**
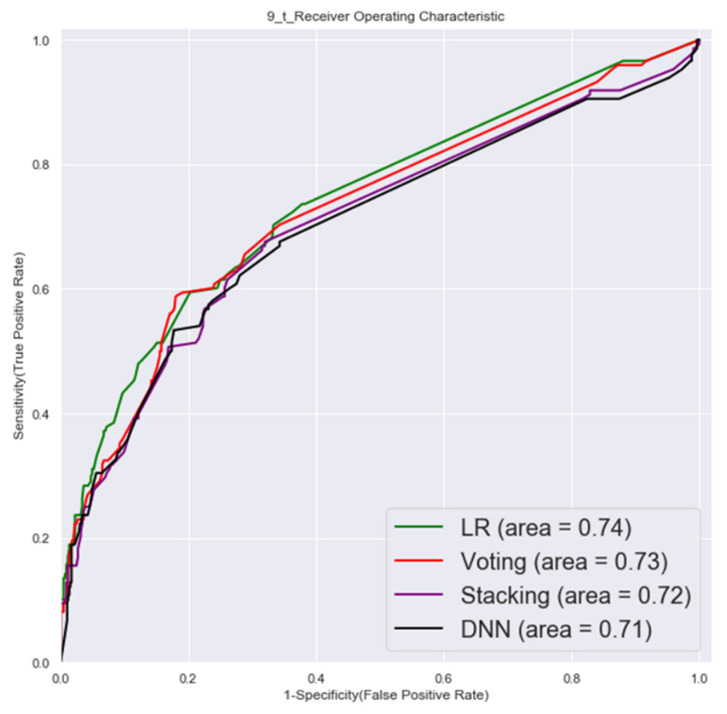
Testing set ROC (third factor combinations).

The ROC of validation set and testing by third factor combinations are shown in Figure 7 and Figure 8.

According to the results, the validation set showed that the voting, stacking, and DNN models tended to be over-optimistic when the factor combinations were more complex (more factors). While a slight improvement was seen in the training phase using SMOTE data augmentation (synthetic training data), a significant improvement was seen when the complexity of the model was simplified (by reducing the number of factors). 

The AUC of the LR model was higher than the other three models in the external testing set for all three of the factor combinations. Sensitivity was best measured by the stacking model for the first factor combinations, and specificity was best measured by the DNN model for the second factor combination. The result of the model that used only nine factors (third factor combinations) was no worse than the other two factor combinations that used more factors for the external testing results. The detailed results are shown in Table 3.

## 4. Discussion

This study developed a pancreatic cancer risk identification prediction model using disease diagnosis records from the NHIRD, and the results were validated through an independent testing set. Pancreatic cancer progresses rapidly, and the average estimated time for progression from stage T1 to stage T4 is 14 months [38], thus, the immediate detection of pancreatic cancer at the resectable stage is the critical goal of early assessment. 

This study constructed a predictive model based on the diagnostic data of 12 months, in order to provide an early warning to patients at the early stage of pancreatic cancer. The results of the before-stated model show that a short history of diseases has the potential for screening and prediction. The AUC of previous models for the early assessment of pancreatic cancer ranged from approximately 0.57 to 0.71. The AUC of this study was higher than that of previous studies and ranged from 0.71 to 0.76, which provides higher accuracy.

A recent study on the development of a prediction model for pancreatic cancer risk screening among the general population [32] proposed a total of 15 factors (abdominal pain, angina pectoris, asthma, atherosclerotic heart disease, gallbladder stones, chest pain, chronic pancreatitis, coronary heart disease, diabetes, emphysema, primary hypertension, family history of pancreatic cancer, jaundice, stroke, and ulcers). The AUC of the training and testing sets were 0.71 and 0.68, respectively. Another model for the assessment of pancreatic cancer applied to health care delivery [29] proposed 16 factors, including the following: acute pancreatitis, chronic pancreatitis, diabetes, dyspepsia, gastritis/peptic ulcer/gallbladder disease, acute cholecystitis, depression, abdominal pain, chest pain, gastrointestinal symptoms, esophageal reflux, jaundice, weight loss/anorexia, nausea/vomiting, fatigue, and tickling, in order to establish a prediction model. The performance analysis of a model with an AUC of 0.61 found that even though the data sources were from different ethnic or national populations, the results of the factors were similar to our study, such as those related to gastrointestinal, gallbladder, pancreatic, diabetes, and chest pain. 

Although not all of the factors were clinically confirmed, the data showed consistency across different regions of the population. In addition, sleep disturbance and hepatitis, which have been less frequently adopted as training factors than those used in previous studies, were found to be among the important key factors in this study. This study presented nine key independent predictors as third combination factors and used a smaller number of predictors than previous studies, and the results show that the model’s AUC performance was higher than that of the previous models for identifying pancreatic cancer in the general population. The detailed results are shown in Table 4.

## 5. Conclusions

This study compared the performances of LR, DNN, stacking, and voting models for pancreatic cancer prediction and constructed a pancreatic cancer prediction model with an accuracy that was higher than that of previous studies. As a reference tool, this diagnostic-based model will help physicians and the public to identify the risk of pancreatic cancer. As this model uses only nine key disease factors, it offers the advantages of low cost and rapid screening. This predictive model will improve awareness of the risk of pancreatic cancer and will give patients with pancreatic cancer a simpler tool for early screening in the golden period when the disease can still be eradicated.

## Figures and Tables

**Table 1:** Demographics of the study group.

Group	Experimental Group	Control Group	Chi-Square
	(With Pancreatic Cancer)	(No Cancer)	*p*-Value
N	738	2214	
Gender			0.517
Male	398 (53.9%)	1194 (53.9%)	
Female	340 (46.1%)	1020 (46.1%)	
Age			1
0~17	1 (0.1%)	1 (<0.1%)	
18~24	14 (1.9%)	28 (1.3%)	
25~34	21 (2.8%)	64 (2.9%)	
35~44	55 (7.5%)	141 (6.4%)	
45~54	95 (12.9%)	234 (10.6%)	
55~64	144 (19.5%)	421 (19.0%)	
65 and above	408 (55.3%)	1325 (59.8%)	
Short-term historical medical information within one year before diagnosis date			
Peptic ulcer, site unspecified (ICD-9 = 533)			<0.001
	138 (18.7%)	126 (5.7%)	
Symptoms involving digestive system (ICD-9 = 787)			<0.001
	51 (6.9%)	63 (2.8%)	
Pancreatitis (ICD-9 = 577)			<0.001
	54 (7.3%)	15 (0.7%)	
Gastritis and duodenitis (ICD-9 = 535)			<0.001
	220 (29.8%)	395 (17.8%)	
Disorders of function of stomach (ICD-9 = 536)			<0.001
	169 (22.9%)	281 (12.7%)	
Functional digestive disorders, not elsewhere classified (ICD-9 = 564)			<0.001
	154 (20.9%)	312 (14.1%)	
Chronic liver disease and cirrhosis (ICD-9 = 571)			<0.001
	120 (16.3%)	134 (6.1%)	
Gastric ulcer (ICD-9 = 531)			<0.001
	51 (6.9%)	63 (2.8%)	
Cholangitis (ICD-9 = 576)			<0.001
	44 (6.0%)	5 (0.2%)	
Duodenal ulcer (ICD-9 = 532)			<0.001
	34 (4.6%)	31 (1.4%)	
Symptoms involving respiratory system and other chest symptoms (ICD-9 = 786)			<0.001
	75 (10.0%)	126 (5.7%)	
Acute and subacute necrosis of liver (ICD-9 = 570)			<0.001
	11 (1.5%)	3 (0.1%)	
Abdominal pain (ICD-9 = 789)			<0.001
	229 (31.0%)	294 (13.3%)	
Symptoms involving head and neck (ICD-9 = 784)			0.001
	72 (9.8%)	325 (14.7%)	
Essential hypertension (ICD-9 = 401)			0.001
	164 (22.2%)	631 (28.5%)	
Acute bronchitis and bronchiolitis (ICD-9 = 466)			0.003
	184 (25%)	403 (18.2%)	
Other and unspecified disorders of the back (ICD-9 = 724)			0.003
	144 (19.5%)	547 (24.7%)	
Heart failure (ICD-9 = 428)			0.004
	9 (1.2%)	60 (2.7%)	
Urticaria (ICD-9 = 708)			0.006
	26 (3.5%)	143 (6.5%)	
Calculus of kidney and ureter (ICD-9 = 592)			0.01
	25 (3.4%)	37 (1.7%)	
Cardiac dysrhythmias (ICD-9 = 427)			0.011
	13 (1.8%)	82 (3.7%)	
Gout (ICD-9 = 274)			0.012
	28 (3.8%)	145 (6.5%)	
Hypertensive heart disease (ICD-9 = 402)			0.013
	66 (8.9%)	279 (12.6%)	
Acute nasopharyngitis (ICD-9 = 460)			0.016
	132 (17.9%)	483 (21.8%)	
Other cellulitis and abscess (ICD-9 = 682)			0.019
	3 (0.4%)	25 (1.1%)	
General symptoms (ICD-9 = 780)			0.022
	159 (21.5%)	422 (19.1%)	
Neurotic disorders (ICD-9 = 300)			0.033
	23 (3.1%)	117 (5.3%)	
Other disorders of pancreatic internal secretion (ICD-9 = 251)			0.034
	6 (0.8%)	5 (0.2%)	
Osteoarthrosis and allied disorders (ICD-9 = 715)			0.034
	76 (10.3%)	298 (13.5%)	
Diabetes mellitus (ICD-9 = 250)			0.035
	155 (21.0%)	368 (16.6%)	
Other forms of chronic ischemic heart disease (ICD-9 = 414)			0.044
	44 (6.0%)	200 (9.0%)	
Acute laryngitis and tracheitis (ICD-9 = 464)			0.045
	64 (8.7%)	264 (11.9%)	
Spondylosis and allied disorders (ICD-9 = 723)			0.054
	46 (6.2%)	182 (8.2%)	
Acute upper respiratory infections of multiple or unspecified (ICD-9 = 465)			0.058
	383 (51.9%)	1248 (56.4%)	
Other disorders of bone and cartilage (ICD-9 = 733)			0.065
	13 (1.8%)	61 (2.8%)	
Other and unspecified disorder of joints (ICD-9 = 719)			0.092
	49 (6.6%)	201 (9.1%)	
Diseases of hard tissues of teeth (ICD-9 = 521)			0.109
	186 (25.2%)	509 (23.0%)	
Urinary tract infection (ICD-9 = 599)			0.133
	51 (6.9%)	195 (8.8%)	
Noninfectious gastroenteritis (ICD-9 = 558)			0.143
	145 (19.6%)	368 (16.6%)	
Diseases of pulp and periapical tissues (ICD-9 = 522)			0.172
	57 (7.7%)	211 (9.5%)	
Allergic rhinitis (ICD-9 = 477)			0.178
	40 (5.4%)	101 (4.6%)	
Acute sinusitis (ICD-9 = 461)			0.184
	76 (10.3%)	221 (10.0%)	
Contact dermatitis and other eczema (ICD-9 = 692)			0.194
	101 (13.7%)	358 (16.2%)	
Cataract (ICD-9 = 366)			0.225
	76 (10.3%)	202 (9.1%)	
Sprains and strains (ICD-9 = 848)			0.234
	40 (5.4%)	153 (6.9%)	
Vertiginous syndromes and other disorders of vestibular system (ICD-9 = 386)			0.234
	9 (1.2%)	41 (1.9%)	
Influenza (ICD-9 = 487)			0.235
	44 (6.0%)	173 (7.8%)	
Disorders of conjunctiva (ICD-9 = 372)			0.257
	144 (19.5%)	539 (24.3%)	
Other disorders of soft tissues (ICD-9 = 729)			0.257
	126 (17.1%)	400 (18.1%)	
Chronic bronchitis (ICD-9 = 491)			0.26
	28 (3.8%)	83 (3.7%)	
Cystitis (ICD-9 = 595)			0.276
	23 (3.1%)	79 (3.6%)	
Other and unspecified arthropathies (ICD-9 = 716)			0.289
	28 (3.8%)	106 (4.8%)	
Cholelithiasis (ICD-9 = 574)			0.312
	16 (2.2%)	24 (1.1%)	
Benign prostatic hyperplasia (ICD-9 = 600)			0.315
	55 (7.5%)	213 (9.6%)	
Bronchitis, not specified as acute or chronic (ICD-9 = 490)			0.343
	17 (2.3%)	72 (3.3%)	
Other disorders of synovium, tendon, and bursa (ICD-9 = 727)			0.387
	37 (5.0%)	134 (6.1%)	
Chronic airways obstruction, not elsewhere classified (ICD-9 = 496)			0.416
	29 (3.9%)	105 (4.7%)	
Pneumonia, organism unspecified (ICD-9 = 486)			0.445
	19 (2.6%)	77 (3.5%)	
Menopausal and postmenopausal disorders (ICD-9 = 627)			0.511
	5 (0.7%)	15 (0.7%)	
Infections of kidney(s) (ICD-9 = 590)			0.518
	3 (0.4%)	10 (0.5%)	
Gingival and periodontal diseases (ICD-9 = 523)			0.628
	224 (30.4%)	667 (30.1%)	
Tooth restoration root (ICD-9 = 525)			0.651
	35 (4.7%)	125 (5.6%)	
Acute tonsillitis (ICD-9 = 463)			0.693
	75 (10.2%)	285 (12.9%)	
Acute appendicitis (ICD-9 = 540)			0.705
	1 (0.1%)	5 (0.2%)	
Other acquired deformity (ICD-9 = 738)			0.748
	6 (0.8%)	16 (0.7%)	
Diseases of the oral soft tissues, excluding lesions specific (ICD-9 = 528)			0.784
	64 (8.7%)	223 (10.1%)	
Acute myocardial infarction (ICD-9 = 410)			0.802
	2 (0.3%)	2 (0.1%)	
Other disorders of cervical region (ICD-9 = 723)			0.909
	21 (2.8%)	55 (2.5%)	
Occlusion of cerebral arteries (ICD-9 = 434)			0.946
	11 (1.5%)	38 (1.7%)	
Acute pharyngitis (ICD-9 = 462)			0.954
	76 (10.3%)	246 (11.1%)	
Angina pectoris (ICD-9 = 413)			0.956
	22 (3.0%)	65 (2.9%)	
Pruritus and related conditions (ICD-9 = 698)			0.958
	27 (3.7%)	74 (3.3%)	
Disorders of lipoid metabolism (ICD-9 = 272)			0.96
	24 (3.3%)	81 (3.7%)	
Fracture of clavicle (ICD-9 = 810)			1
	2 (0.3%)	4 (0.2%)	

**Table 2:** The results of stepwise regression.

Backward Elimination			
Disease	*p*-Value	Disease	*p*-Value
Abdominal pain	1.16 × 10^−7^ ***	Gout	0.038517 *
Peptic ulcer, site unspecified	2.06 × 10^−14^ ***	Functional digestive disorders, not elsewhere classified	0.019337 *
Symptoms involving digestive system	3.35 × 10^−5^ ***	Neurotic disorders	0.069057
Gastritis and duodenitis	0.000162 ***	Disorders of conjunctiva	0.090836
Disorders of function of stomach	3.88 × 10^−5^ ***	Heart failure	0.090678
Chronic liver disease and cirrhosis	2.51 × 10^−12^ ***	Essential hypertension	0. 054,498
General symptoms	0.000860 ***	Other forms of chronic ischemic heart disease	0.068356
Cholangitis	1.03 × 10^−14^ ***	Other and unspecified disorders of the back	0.082334
Pancreatitis	4.14 × 10^−9^ ***	Duodenal ulcer	0.059147
Symptoms involving head and neck	0.005121 **	Calculus of kidney and ureter	0.067626
Symptoms involving respiratory system and other chest symptoms	0.007710 **	Acute nasopharyngitis	0.136144
Urticaria	0.002506 **	Acute laryngitis and tracheitis	0.123336
Other cellulitis and abscess	0.003819 **	Gastric ulcer	0.141536
Acute bronchitis and bronchiolitis	0.021931 *	* Hypertensive heart disease	0.153331
Cardiac dysrhythmias	0.032659 *	Osteoarthrosis and allied disorders	0.13224
Acute and subacute necrosis of liver	0.010382 *	Other disorders of pancreatic internal secretion	0.132718
Diabetes mellitus	0.017619 *	Significant codes: <0.001 ‘***’ 0.001 ‘**’ 0.01 ‘*’	

**Table 3:** Model performance comparison.

First Combination (32 Factors)	Validation Set				Testing Set (External Set)			
	AUC	Accuracy	Sensitivity	Specificity	AUC	Accuracy	Sensitivity	Specificity
LR	0.78	0.73	0.7	0.74	0.76	0.73	0.7	0.74
Voting	0.87	0.77	0.76	0.77	0.75	0.7	0.7	0.71
Stacking	0.85	0.77	0.71	0.82	0.74	0.7	0.74	0.7
DNN	0.89	0.82	0.78	0.86	0.73	0.65	0.65	0.66
**Second Combination** **(19 Factors)**	**Validation Set**				**Testing Set (External Set)**			
	**AUC**	**Accuracy**	**Sensitivity**	**Specificity**	**AUC**	**Accuracy**	**Sensitivity**	**Specificity**
LR	0.78	0.7	0.7	0.7	0.77	0.7	0.7	0.7
Voting	0.83	0.73	0.74	0.72	0.76	0.71	0.71	0.71
Stacking	0.82	0.73	0.74	0.73	0.73	0.7	0.7	0.72
DNN	0.82	0.73	0.72	0.74	0.71	0.7	0.64	0.76
**Third Combination** **(9 Factors)**	**Validation Set**				**Testing Set (External Set)**			
	**AUC**	**Accuracy**	**Sensitivity**	**Specificity**	**AUC**	**Accuracy**	**Sensitivity**	**Specificity**
LR	0.77	0.7	0.7	0.7	0.74	0.68	0.7	0.67
Voting	0.76	0.7	0.7	0.7	0.73	0.67	0.7	0.66
Stacking	0.77	0.7	0.7	0.7	0.72	0.68	0.68	0.68
DNN	0.77	0.7	0.7	0.71	0.71	0.68	0.7	0.67

**Table 4:** Model comparison with previous studies.

Research Team	Factor	Algorithm	Data Resource	Data Period	Performance
This study	Abdominal pain, peptic ulcers, flatulence, gastritis, abnormal gastric function, hepatitis, sleep disorders, cholangitis, pancreatitis (9 factors)	Logistic regression	NHIRD	Before diagnosis, within 12 months	Validation Set: 0.77 Testing Set: 0.74
Limor Appelbaum 2021 [28]	Abdominal pain, angina pectoris, asthma, atherosclerotic heart disease, gallbladder stones, chest pain, chronic pancreatitis, coronary heart disease, diabetes mellitus, emphysema, primary hypertension, family history of pancreatic cancer, jaundice, stroke, ulcers (15 factors)	Logistic regression	Electronic health record at Boston Hospital	Before diagnosis, 6–12 months	0.68–0.75
Aileen Baecker 2019 [29]	Acute pancreatitis, chronic pancreatitis, diabetes mellitus, dyspepsia, gastritis/peptic ulcer/gallbladder disease, acute cholecystitis, depression, abdominal pain, chest pain, gastrointestinal symptoms, esophageal reflux, jaundice, weight loss/anorexia, nausea/vomiting, fatigue, tickling disorder (16 factors)	Logistic regression	SEER database	Before diagnosis, within 15 months	0.68
Alison P Klein 2013 [30]	Smoking, alcohol consumption, diabetes, obesity, family history of pancreatic cancer, non-O ABO genotype (6 factors)	Absolute risk regression	PanScan consortium		0.58–0.61

## Data Availability

The data presented in this study are available on request from the corresponding author.
